# Risk Factors of Portal Vein Thrombosis after Devascularization Treatment in Patients with Liver Cirrhosis: A Nested Case-Control Study

**DOI:** 10.1155/2020/9583706

**Published:** 2020-08-27

**Authors:** Shenxin Lu, Guohua Hu, Shiyao Chen, Jian Wang

**Affiliations:** ^1^Liver Cancer Institute, Zhongshan Hospital, Fudan University and Key Laboratory of Carcinogenesis and Cancer Invasion, Ministry of Education, Shanghai, China; ^2^Department of Gastroenterology and Hepatology, Zhongshan Hospital, Fudan University, Shanghai, China; ^3^Department of General Surgery, Zhongshan Hospital, Fudan University, Shanghai, China; ^4^Evidence-Based Medicine Center, Fudan University, Shanghai, China

## Abstract

**Methods:**

We retrospectively reviewed medical records from cirrhosis patients who underwent devascularization for the treatment of portal hypertension in our hospital between January 1, 2008, and December 20, 2014. Patients were followed up to investigate the PVT incidence at different times after surgery. Patients were divided into two groups (PVT, no PVT), and the risk factors for PVT after surgery were determined.

**Results:**

Until October 16, 2015, the median follow-up time of the 124 patients enrolled into this study was 41.43 months (range, 5.47–95.30 months). 61 patients had perioperative PVT, and 21 (16.94%) patients had PVT diagnosed during the follow-up period. Those who had lower preoperative white blood cell counts, larger preoperative portal vein trunk diameter, and no gastric varices were more likely to have perioperative thrombosis. In those without perioperative PVT, a history of hypertension, higher grade of splenomegaly, and higher preoperative levels of creatinine were independent predictors of PVT occurrence during the follow-up period.

**Conclusions:**

The risk factors for perioperative PVT in cirrhotic patients after devascularization were lower preoperative white blood cell count and larger portal vein trunk diameter, with no gastric varices. A history of hypertension, a larger spleen, and higher preoperative creatinine level are independent predictors of PVT during follow-up after surgery in patients without perioperative PVT.

## 1. Introduction

Portal vein thrombosis (PVT) is complete or partial blood flow obstruction in the main trunk and branches of the portal vein, which is common in patients with cirrhosis.

In patients with cirrhotic portal hypertension, those with PVT have a worse prognosis and a higher rebleeding rate after treatment [1]. Studies have shown that PVT is an independent risk factor for treatment failure in acute esophageal variceal bleeding [2]. However, most clinical PVT has an absence of obvious symptoms before complications, so it is difficult to diagnose and treat in a timely manner. Thus, early diagnosis and proper treatment are important for patient prognosis because of the nonspecific clinical manifestations and adverse effects [3]. Determining the risk factors for PVT in patients with liver cirrhosis can provide a reference for prevention, diagnosis, and treatment to improve patient prognosis.

In our previous study, the rebleeding rate is higher in the patients whose PVT developed during follow-up (47.6%) than those with perioperative PVT (9.8%) (*p* = 0.015) [4]. In China, devascularization and splenectomy are commonly used measures to prevent rebleeding caused by portal hypertension (secondary prevention). The incidence of PVT in the general cirrhosis population is 10%–25% [5, 6], and the incidence of PVT after splenectomy was 4.8%–51.5% [7–10].

There are many factors related to PVT occurrence during short-term follow-up after splenectomy such as a wider portal vein, low preoperative white blood cell count, high postoperative platelet count, prolonged prothrombin time, pericardial devascularization, and a larger spleen size [11, 12].

The risk factors for PVT were different in different studies, and it has been suggested that an increase in morbidity may result from surgical procedures rather than from splenectomy [8, 13]. Additionally, there have been no studies on the long-term occurrence of PVT and its risk factors after splenectomy.

In this study, we retrospectively explored the risk factors for PVT in patients with liver cirrhosis after splenectomy and devascularization surgery.

## 2. Methods

This is a nested case-control study. The study retrospectively analyzed the medical record from cirrhosis patients 18 years or older who underwent devascularization to prevent esophageal variceal rebleeding in our hospital between January 1, 2008, and December 20, 2014. The exclusion criteria were as follows: (1) age < 18 years; (2) noncirrhosis patients; (3) patients who had previously undergone splenectomy, splenic embolization, or shunt surgery (including surgical shunt and transjugular intrahepatic portosystemic shunt (TIPS)); (4) no surgery resulting from severe abdominal adhesions; (5) patients with postoperative intraoperative bleeding resulting from improper vascular ligation; (6) patients with emergency interruption surgery; (7) patients with preoperative or perioperative portal imaging information; (8) patients with PVT before surgery; (9) patients who died perioperatively; (10) patients with liver cancer or other malignancies; (11) history of no gastrointestinal bleeding; or (12) patients who are lost to follow-up after discharge.

### 2.1. Definitions

*PVT*: complete or partial thrombus that occurs in the main trunk, left branch, or right branch of the portal vein with or without extending to splenic vein or superior mesenteric vein, based on ultrasound, CTA, or MRA. If one thrombosis was present at more than one examination, PVT was diagnosed. All the patients with liver tumors were examined using magnetic resonance imaging to exclude tumor thrombus.

*Perioperative thrombosis*: thrombosis found day 7 after routine ultrasonography examination.

*Perioperative death*: death that occurred during the first week after surgery. Perioperative death was also defined if the patient was not discharged in the first week because of complications related to surgery and died before the complications were resolved.

*Grade of esophageal varices*: esophageal varices were classified into light, medium, and severe based on the definition specified by the Varicosity, Committee Of Esophageal Association, Society Of Digestive Endoscopy, 2009 [14].

*Spleen volume*: spleen volume = 30 + 0.58 (length × width × thickness) [15]. The spleen length, width, and thickness were obtained via postoperative spleen pathology.

*Grade of splenomegaly*: divided into three grades (mild, moderate, and severe) based on palpation (mild = the spleen is less than 2 cm over the ribs during deep breathing; moderate = during deep breathing, the spleen was more than 2 cm above the ribs but did not extend past the umbilical level; severe = the spleen extends past the navel horizontal line or over the front midline during deep breathing).

### 2.2. Data Collection

Patients were enrolled based on the inclusion and exclusion criteria and medical history information that was collected retrospectively. The follow-up period ended October 16, 2015. Patients underwent an imaging examination of the portal system on day 7, 6 months after the surgery, and every 6 months thereafter. Medical history, physical examination results, imaging data, and laboratory examination results including preoperative, perioperative, and follow-up information were collected retrospectively using the hospital's medical records, and attempts were made to complete missing or uncertain items by telephone follow-up.

Patients were divided into two groups based on the perioperative and postdischarge follow-up period. Patients without preoperative thrombosis were divided into case and control groups based on whether they had perioperative thrombosis, and risk factors for perioperative thrombosis were analyzed. Patients without perioperative thrombosis were divided into case and control groups based on whether they developed PVT during follow-up, and risk factors for follow-up PVT were analyzed.

### 2.3. Statistical Methods

The SPSS19.0 (SPSS Inc., Chicago, IL, USA) was used to analyze the study data. Univariate analysis was used first to identify possible risk factors. Continuous variables with normal distribution are expressed as the mean ± standard deviation. Student's *t*-test was used to compare between groups. Continuous variables with nonnormal distribution are expressed as the mean ± quartiles (interquartile range (IQR)), and nonparametric tests were performed between groups. Normal distribution was assessed using the Kolmogorov–Smirnov method (K–S method). Categorical variables are expressed in terms of constituent ratios. Disorderly classification variables were analyzed using the chi-square test (when 1/5 of the lattice theoretical frequency was <5, or the lattice of the theoretical frequency was <1 using Fisher's exact test). The rank data were compared using the rank sum test. A multivariate analysis was performed using logistic regression. *p* < 0.05 was considered statistically significant. For univariate analysis, *p* < 0.2 was considered statistically significant.

## 3. Ethical Approval

All procedures performed in studies involving human participants were in accordance with the ethical standards of the institutional research committee (Ethics Committee of Zhongshan Hospital Fudan University + B2015-13R) and with the 1964 Helsinki Declaration and its later amendments or comparable ethical standards.

## 4. Results

**Figure 1:**
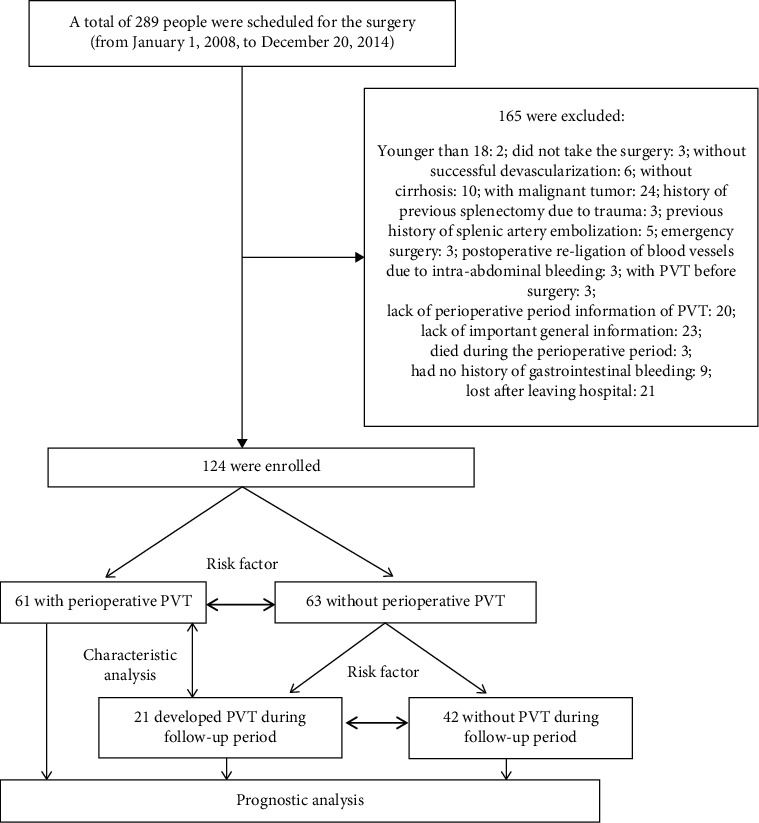
Research flowchart.

From January 1, 2008, to December 20, 2014, 289 patients were admitted to our department for general surgery because of portal hypertension. All these patients were taken to surgery because of severe hypersplenism or endoscopic treatment failure. Among these patients, 165 were excluded based on the exclusion criteria, and 124 patients (42.91%) were enrolled into the study. The enrollment process is shown in Figure 1.

The average age was 49.83 years (range, 18–77 years), comprising 83 males (66.94%) and 41 females (33.06%). The patients underwent the following procedures after admission to the hospital: 98 (79.03%) underwent splenectomy combined with combined devascularization, and 26 (20.97%) underwent splenectomy combined with pericardial devascularization.

### 4.1. Overall Follow-Up and Retreatment

The average follow-up time was 42.73 months for all patients until October 16, 2015, with a median follow-up of 41.43 months (range, 5.47–95.30 months). A total of 22 (17.7%) patients had rebleeding and 10 (8.1%) patients died during follow-up (including four patients who developed rebleeding). A life table was used to obtain the nonbleeding and survival rates for all patients, and the 1-, 3-, and 5-year rebleeding-free rate was 92%, 83%, and 69%, respectively. The 1-, 3-, and 5-year survival rate was 96%, 92%, and 85%. A total of 25 patients received other special treatments during follow-up, including 23 patients who underwent endoscopic treatment, one of whom was treated using balloon-occluded retrograde transvenous obliteration (BRTO) and two who were treated using TIPS.

### 4.2. Thrombosis Development

There were 61 (49.2%) patients who developed thrombosis during the perioperative period. Of the 63 patients who did not develop thrombosis during the perioperative period, 21 (16.94%) had thrombosis during the follow-up period and 42 (33.87%) had no thrombosis during follow-up.

In patients without perioperative thrombosis, if the platelet count was ≥350 × 10^9^/L, aspirin was taken for 1 month postoperatively and they received no antiplatelet therapy during long-term follow-up. All patients with thrombosis (including perioperative thrombosis and thrombosis observed during follow-up) were taking aspirin and dipyridamole after the thrombosis was discovered, and it continued until thrombosis recanalization.

Four patients with perioperative thrombosis and one patient with follow-up PVT had taken anticoagulants during follow-up, and the remaining patients did not receive anticoagulant therapy.

### 4.3. Analysis of the Risk Factors Associated with Perioperative Thrombosis

Patients were divided into two groups based on whether thrombosis occurred during the perioperative period, and the indicators were analyzed by univariate analysis (Table 1). The statistically significant (*p* < 0.2) indicators of the univariate analysis were included in the multivariate logistic regression.

Finally, the preoperative leukocyte count, postoperative portal vein diameter, and the presence of varicose veins were independent factors for perioperative thrombosis (*p* < 0.05, Table 2). Among them, a wider postoperative portal vein diameter was a risk factor, and a higher leukocyte count before surgery and the presence of varicose veins were protective factors.

### 4.4. Analysis of Risk Factors for Thrombosis during Follow-Up

Of the 63 patients who did not develop thrombosis during the perioperative period, 21 had thrombosis during the follow-up procedure, and the median time to thrombosis was 24.3 months (range, 0.73–87.63 months). Forty-two patients had no thrombosis during the follow-up period.

Among patients who did not develop thrombosis during the perioperative period, those who developed cancer during follow-up were excluded. The remaining patients underwent a nested case-control study based on whether thrombosis occurred during follow-up to explore the risk factors associated with thrombosis, except for tumors. Univariate analysis was also used to the possible indicators (Table 3). Factors with *p* < 0.2 in a univariate analysis were included in the multivariate unconditional logistic regression model. The indexes of hypertension, spleen enlargement, and preoperative creatinine level were significantly different (*p* < 0.05), the odds ratio was greater than 1, and the independent risk factors were identified (Table 4).

## 5. Discussion

Endoscopic treatment, surgery, and TIPS are all options for secondary prevention for rebleeding. Although with the development of endoscopy and interventional technology, more patients may choose endoscopic treatment or TIPS, devascularization is still being performed in many centers.

The rebleeding rate and mortality after devascularization in patients with liver cirrhosis varied in previous studies. The 3-year survival rate of patients who underwent traditional devascularization was 95.52% according to Yang et al. [16], while Lu et al. [17] reported a 5-year survival rate of 86% and a rebleeding-free rate of 84.8%. Du et al. [18] showed that the 1-year, 3-year, and 5-year survival rate was 96.7%, 83.3%, and 73.3%, respectively. None of the 54 patients in the study by Zheng et al. [19] had a rebleeding event during the follow-up period (3–36 months), and in another study by Zhou et al. [20], no deaths were reported in a mean of 25 months after surgery. Besides the included population, another probable reason for the variability in the survival and rebleeding rates obtained from previous studies is the difference in surgical methods. In this study, the included patients had a 1-year, 3-year, and 5-year rebleeding-free rate of 92%, 83%, and 69% and a 1-year, 3-year, and 5-year survival rate of 96%, 92%, and 85%, respectively, which was consistent with the results of previous studies.

PVT is a common complication after devascularization and splenectomy. The incidence of PVT after surgery varies in current studies. The incidence of PVT in patients with cirrhosis during the natural course of the disease is about 10%–25% [5, 6], and the incidence of PVT after surgery was 4.8%–51.5% in the absence of cirrhosis [8]. However, the incidence of PVT after surgery was 30.1–47.8% in patients with cirrhosis [13, 21], which occurred in a median of 6 days (range, 3–11 days) from surgery to discovery of asymptomatic thrombosis [13]. In this study, 66.13% (82/124) of the patients developed PVT after the devascularization, and 49.19% (61/124) of the patients developed PVT within 7 days after the surgery, which is consistent with previous studies.

The higher incidence of PVT in splenectomy patients in the short term after surgery is also related to these three factors. Previous studies showed that the factors potentially related to PVT in the short term after splenectomy were the preoperative portal vein diameter, preoperative leukocyte count, postoperative increase in the platelet count, prothrombin time prolongation, surgical approach including pericardial devascularization, and spleen size [11, 12]. Platelet, erythrocyte, and leukocyte counts after splenectomy increased rapidly over a short time, and the blood was in a hypercoagulable state [22, 23]. Therefore, some studies have suggested that preoperative low platelet and leukocyte counts are a risk factor for postoperative thrombosis [12]. Kinjo et al. showed that the splenic vein diameter was negatively correlated with portal blood flow velocity, and the portal vein blood flow rate decreased by 49% in patients with postoperative PVT [12]. Other studies considered a wide preoperative portal vein diameter [11], splenic vein diameter [12], spleen thickness [24], and spleen volume [25] to be associated with slower portal blood flow, and they may also be risk factors for postoperative thrombosis. There are also a large number of vascular stumps after devascularization. However, turbulence is easy to cause, and the surgery caused additional damage to the vascular endothelium, resulting in a higher risk of developing thrombosis. In our study, a wide range of portal vein diameters and a lower white blood cell count were considered to be risk factors in a multivariate analysis, which is consistent with previously published studies. Additionally, all patients in our study underwent splenectomy and pericardial devascularization surgery, which was consistent with Yang Zhen's method [26], while combined devascularization surgery blocked blood vessels in the gastric basement and submucosal layers. Based on pericardial devascularization, pericardial devascularization is thought to be a factor that may affect the development of thrombosis postoperatively. In our study, patients with no gastric varices were more likely to have perioperative thrombosis, which may be because the patients with gastric varices are more often to have pericardial devascularization.

Additionally, 33.33% (21/63) of the patients who did not develop thrombosis during the perioperative period were followed up, and 33.33% (21/63) of them developed PVT during the follow-up period. There have been few studies on the patients who did not develop thrombosis during the perioperative period, and we found that the risk factors for perioperative PVT and follow-up PVT were different. We suggest that thrombosis occurring during the follow-up period is less affected by the surgery and may more closely resemble the PVT that develops in the natural disease course of cirrhosis, which occurs as a result of an imbalance in the anticoagulation-coagulation system.

Hypertension is a risk factor for deep vein thrombosis. In cirrhosis patients, endothelin (ET) and nitric oxide (NO) levels in peripheral blood or intrahepatic circulation were different from those in patients without cirrhosis. Hypertensive people have an increased peripheral blood ET level and a decreased NO level compared with those without hypertension, which is also an unbalanced state [27]. There is no evidence that the imbalance between ET and NO levels in hypertensive patients has a significant effect on portal venous blood flow. Additional data on whether these hypertensive patients had taken nonselective beta-blockers (NSBBs) during follow-up were not obtained. It is possible that thrombosis development may have been related to NSBBs rather than to the high blood pressure. It is not possible to form a conclusion based on the current information.

The incidence of PVT was associated with spleen size in previous studies, but the risk factors remained controversial. Spleen volume was a risk factor for PVT (*p* < 0.05) in a study by Iida et al. [25], while Li et al. showed that spleen volume was independent of PVT development (*p* = 0.925) [11]. Kinjo et al. showed that, although the spleen weight was statistically significant in a univariate analysis, it was not associated with thrombosis development in a multivariate analysis [12]. Chen et al. showed that spleen thickness was a risk factor for thrombosis (*p* < 0.01) [24], and Jiang et al. found that the spleen long diameter was not a risk factor for PVT (*p* = 0.298). This difference may result from different populations and different measurement methods [21]. In Kinjo et al.'s study, 36% of the patients also had hepatic carcinoma and the measurement method used was pathological spleen size [12]. Li et al. also did not exclude patients with hepatic carcinoma and also used the pathological spleen size [11], while Chen et al. excluded hepatic carcinoma patients and used ultrasound results as an indicator of spleen size [24]. Additionally, Jiang et al. did not indicate upon what the spleen diameter was based, and different methods were not compared in the same article [21]. In our study, there was no statistical difference in spleen length, width, thickness, and volume, but the classification of palpation in the spleen was a risk factor for thrombosis, which may result from different sized patients who have different absolute spleen sizes. The results showed a large standard deviation in the pathological spleen size, indicating more obvious differences within the group and there was no difference between groups that may be affected by the smaller sample size. Palpation of the spleen was less affected by the patient's figure because it is based on the relative degree of spleen enlargement. It is unknown whether the pathological results have clinical significance for the development of PVT, and this requires a large-scale study to be conducted.

Hepatorenal syndrome is a possible complication in patients with decompensated cirrhosis. Additionally, thrombopoietin is mainly produced in the kidney, so changes in renal function may affect platelet number and function, while many scholars believe that thrombosis in patients after splenectomy is a result of changes in platelet function [28]. The reason that increased creatinine was a risk factor for thrombus formation is because of the influence of creatinine on platelet function in patients after splenectomy, or the increase in creatinine reflects the effect of deteriorating liver function on renal function. Further studies to investigate this are also required.

Because damage was greatly reduced, platelet levels in patients with splenectomy were significantly increased after surgery. The role of platelets in the development of PVT in cirrhosis patients is controversial. S. He and F. He found that platelet volume and average platelet volume were good predictors of thrombosis after splenectomy [29]. However, Girleanu et al. analyzed the platelet volume in cirrhosis patients with and without PVT and they found that the mean platelet volume and platelet width contribute to thrombosis in cirrhosis patients rather than the number of platelets [30]. In this study, the number of platelets did not correlate with the incidence of PVT during the follow-up period, suggesting that the functional changes in platelets maybe play a stronger role in the pathogenesis of portal thrombosis after splenectomy compared with platelet counts.

This study has some limitations. First, this study is a retrospective study. Information about treatment, development of thrombosis and rebleeding, and death were obtained via follow-up by phone, so there may be a memory bias, and there are some deficiencies in the data integrity. For example, in patients with hypertension, the details about taking antihypertensive drugs, the duration of hypertension, and blood pressure control were not obtained, which may have affected the results. Second, 15.67% of the patients were lost to follow-up during the follow-up period in this study, which is a high loss rate. Some patients were excluded because of a lack of data, which may lead to choice bias. Third, the study sample size is small and it is a single-center study. However, surgical and perioperative management both have an effect on the patient's prognosis and PVT development. Additionally, the study included patients with a variety of liver cirrhosis causes, but all these patients underwent prophylactic surgery for hemorrhage and patients with hepatocellular carcinoma were excluded. Therefore, the conclusions may not be generalizable to the general public. Fourth, patients diagnosed with thrombosis underwent surgery using ultrasound, CTA, or magnetic resonance during follow-up. There was no comparison between each of these methods. However, different methods may have different diagnostic sensitivities, which may produce some diagnostic bias. Additionally, some patients had an earlier or irregular follow-up because of bleeding and other events that may affect the timing of thrombosis. Fifth, in exploring the risk factors for thrombosis during follow-up, tumors were considered to be a risk factor for deep venous thrombosis because of the hypercoagulable state caused by the tumor; because this is different from the risk factors for thrombosis in nontumor patients, patients who developed a tumor during the follow-up period were excluded. This may also affect the generalizability of the results.

A combination of different risk factors may lead to the development of the PVT after devascularization in cirrhosis patients. Patients who developed thrombosis during follow-up had different risk factors compared with those whose PVT occurred perioperatively and those without thrombosis. Therefore, patients without thrombosis perioperatively should be followed up for thrombosis, and thus, thrombosis may be actively prevented in patients with risk factors. However, additional large-scale prospective studies are needed to verify these conclusions.

## 6. Conclusion

Risk factors for perioperative PVT and PVT that occurs in the natural history of cirrhotic patients after surgery are different. Patients with a lower preoperative white blood cell count, a larger portal vein trunk diameter, and no gastric varices are more likely to have perioperative thrombosis. A history of hypertension, a larger spleen, and a higher creatinine level preoperatively are independent predictors for the occurrence of PVT during the follow-up period after surgery in patients without perioperative PVT.

## Figures and Tables

**Table 1:** Univariate analysis of risk factors for perioperative thrombosis.

Factor		Without PVT (*n* = 63)	PVT (*n* = 61)	*p*
Age		52.65 ± 11.78	46.92 ± 11.61	**0.007**
Meld score		21.74 ± 4.04	21.98 ± 3.50	0.723
The time from the first hemorrhage to surgery (years)∗		0.4 (0.2-0.9) 0.96 ± 1.97	0.4 (0.2-1.15) 1.14 ± 1.83	0.633 (0.604)
Spleen length (cm)∗		**15 (14-18)** 15.62 ± 2.71	**17 (15-19)** 16.94 ± 3.08	**0.015** **(0.012)**
Spleen width (cm)∗		**10 (10-12)** 10.53 ± 2.28	**11 (10-13)** 11.33 ± 2.10	**0.029** **(0.044)**
Spleen thickness (cm)∗		**6 (5-6.5)** 5.97 ± 1.84	**6 (5-8)** 6.48 ± 1.83	**0.044** **(0.123)**
Red blood cells (×10^12^/L)		3.5792 ± 0.61	3.6215 ± 0.61	0.7
Hemoglobin (g/L)		95.62 ± 20.87	92.02 ± 21.99	0.251
White blood cells (×10^9^/L)		2.71 ± 1.16	2.05 ± 0.93	**0.001**
Platelets (×10^9^/L)∗		57 (36-75) 63.14 ± 34.49	47 (35.5-71) 55.39 ± 25.29	0.312 (0.157)
Total bilirubin (*μ*mol/L)		16.54 ± 7.41	17.47 ± 6.77	0.476
Combined bilirubin (*μ*mol/L)∗		**5.9 (4.9-8.3)** 7.18 ± 3.54	**7.1 (5.6-9.7)** 8.09 ± 3.88	**0.072** **(0.174)**
Albumin (g/L)		35.73 ± 3.85	37.15 ± 3.65	**0.038**
Alanine aminotransferase (U/L)∗		25 (18-37) 31.33 ± 21.50	26 (19.5-36) 31.05 ± 17.73	0.982 (0.936)
Aspartate aminotransferase (U/L)∗		32 (25.72-43.5) 37.18 ± 18.50	31.0 (23.0-43.5) 36.43 ± 16.22	0.560 (0.811)
Creatinine (*μ*mol/L)∗		69 (59-80) 75.40 ± 29.17	72 (59-82) 74.57 ± 30.17	0.690 (0.877)
Urea (mmol/L)∗		4.7 (3.8-5.7) 5.49 ± 4.21	5 (4.15-5.95) 5.12 ± 1.44	0.656 (0.51)
Uric acid (*μ*mol/L)		317.3 ± 99.12	298.23 ± 61.02	**0.198**
Activated partial thromboplastin time (s)		35.56 ± 6.08	37.14 ± 5.35	**0.13**
International normalization ratio		1.27 ± 0.14	1.26 ± 0.13	0.427
Thrombin time (s)		18.25 ± 1.33	18.10 ± 2.55	0.665
Prothrombin time (s)∗		14.3 (13.6-15.6) 14.58 ± 1.57	14.5 (13.75-15.65) 14.10 ± 3.12	0.517 (0.345)
Fibrinogen (mg/dL)		169.6 ± 42.18	164.33 ± 41.14	0.485
Portal vein diameter (mm)∗		**11.8 (10-13)** 11.621 ± 1.821	**12.5 (11.0-14.0)** 12.89 ± 2.03	**0.001** **(<0.001)**
Portal vein blood flow velocity (m/s)		0.19 ± 0.07	0.18 ± 0.06	0.535
Postoperative platelets (×10^9^/L)		289.82 ± 124.28	312.68 ± 128.99	0.321
Spleen volume (cm^3^)∗		**552.0 (419.76-762.83)** 648.07 ± 392.84	**720.2 (535.73-1028.76)** 796.11 ± 406.45	**0.005** **(0.041)**
Previous history of endoscopic therapy	No	47 (74.6%)	51 (83.6%)	0.218
	Yes	16 (25.4%)	10 (16.4%)
Child-Pugh grade	A	**46 (73.0%)**	**52 (85.2%)**	**0.094**
	B+C	**17 (27.0%)**	**9 (14.8%)**	
History of abdominal surgery	No	57 (90.5%)	58 (95.1%)	0.492
	Yes	6 (9.5%)	3 (4.9%)	
Combined devascularization	No	**18 (28.6%)**	**8 (13.1%)**	**0.035**
	Yes	**45 (71.4%)**	**53 (86.9%)**	
Peripheral blood vessel dissection	No	**45 (71.4%)**	**53 (86.9%)**	**0.035**
	Yes	**18 (28.6%)**	**8 (13.1%)**
Hypertension	No	56 (88.9%)	58 (95.1%)	0.324
	Yes	7 (11.1%)	3 (4.9%)
Diabetes	No	54 (85.7%)	55 (90.2%)	0.447
	Yes	9 (14.3%)	6 (9.8%)
Smoking	No	**56 (88.9%)**	**47 (77.0%)**	**0.079**
	Yes	**7 (11.1%)**	**14 (23.0%)**
History of blood transfusion	No	34 (54.0%)	31 (50.8%)	0.726
	Yes	29 (46.0%)	30 (49.2%)
Grade of splenomegaly∗∗	0	21 (33.3%)	9 (14.8%)	0.440
	I	14 (22.2%)	21 (34.4%)	(0.007)
	II	6 (9.5%)	16 (26.2%)	
	III	22 (34.9%)	15 (24.6%)	
Grade of esophageal varices∗∗	No	1 (1.6%)	0 (0%)	0.593
	Mild	1 (1.6%)	1 (1.6%)	(0.737)
	Moderate	9 (14.3%)	7 (11.5%)	
	Severe	52 (82.5%)	49 (80.3%)	
With gastric varices	No	**21 (33.3%)**	**33 (54.1%)**	**0.020**
	Yes	**42 (66.7%)**	**28 (45.9%)**	
Ascites	No	47 (74.6%)	43 (70.5%)	0.608
	Yes	16 (25.4%)	18 (29.5%)
Hepatic encephalopathy	No	62 (98.4%)	60 (98.4%)	1.0
	Yes	1 (1.6%)	1 (1.6%)	
Posthepatitic cirrhosis	No	23 (36.5%)	18 (29.5%)	0.407
	Yes	40 (63.5%)	43 (70.5%)

∗*p* < 0.05 using the K–S test for data that were not normally distributed. A nonparametric test was used to compare the groups. The results obtained using *t*-tests are also included. ∗∗Graded variables. Rank sum tests were used to compare groups, and the chi-square test results are also included.

**Table 2:** Multivariate analysis of risk factors for perioperative thrombosis (logistic regression: forward-LR).

Risk factors	Regression coefficients	*p*	OR	95% confidence interval for OR
Preoperative white blood cells	-0.777	0.002	0.460	0.284-0.744
Diameter of portal vein trunk preoperatively	0.330	0.005	1.391	1.102-1.757
With gastric varices	-1.335	0.003	0.263	0.108-0.638
Constant	-1.533	0.339	0.216	

**Table 3:** Univariate analysis of risk factors for thrombosis during follow-up.

Factor		PVT (*n* = 19)	Without PVT (*n* = 35)	*p*
Age (years old)		52.63 ± 12.84	53.23 ± 11.13	0.859
First bleeding time from surgery (years)∗		0.4 (0.17~0.80) 0.94 ± 1.86	0.4 (0.20-1.00) 1.13 ± 2.28	0.649 (0.766)
Meld score		22.60 ± 4.13	20.86 ± 3.72	**0.120**
Spleen length (cm)		16.21 ± 3.31	15.00 ± 2.36	**0.126**
Spleen width (cm)		10.42 ± 2.41	10.21 ± 2.08	0.742
Spleen thickness (cm)∗		6 (5~6.5) 6.105 ± 2.38	6 (5-6) 5.82 ± 1.42	0.802 (0.594)
Red blood cell (×10^12^/L)		3.58 ± 0.65	3.58 ± 0.58	0.987
Hemoglobin (g/L)		96.05 ± 24.052	96.31 ± 19.57	0.903
White blood cell (×10^9^/L)		2.63 ± 1.20	2.91 ± 1.17	0.423
Platelet (×10^9^/L)		65.21 ± 40.667	66.63 ± 33.94	0.968
Total bilirubin (*μ*mol/L)		16.77 ± 6.41	16.19 ± 7.64	0.779
Conjugated bilirubin (*μ*mol/L)∗		6.7 (5.4~10.5) 7.47 ± 3.28	5.8 (4.7~7.8) 6.97 ± 3.57	0.394 (0.614)
Albumin (g/L)		36.95 ± 3.24	35.03 ± 3.78	**0.067**
Alanine aminotransferase (U/L)		33.63 ± 21.454	30.94 ± 23.87	0.684
Aspartate aminotransferase (U/L)		36.74 ± 19.550	37.59 ± 19.86	0.881
Creatinine (*μ*mol/L)		80.579 ± 23.5686	68.63 ± 12.86	**0.051**
Urea (mmol/L)∗		5.2 (3.4~7.1) 5.1 ± 2.01	4.7 (3.8~5.3) 5.62 ± 5.43	0.650 (0.687)
Uric acid (*μ*mol/L)		337.95 ± 100.568	310.00 ± 109.48	0.361
Activated partial thromboplastin time (s)		34.44 ± 6.28	36.33 ± 5.92	0.287
International normalization ratio		1.25 ± 0.16	1.28 ± 0.14	0.395
Thrombin time (s)		18.08 ± 1.47	18.23 ± 1.19	0.694
Prothrombin time (s)		14.16 ± 1.68	14.82 ± 1.65	**0.169**
Fibrinogen (mg/dL)		175.17 ± 50.625	168.97 ± 39.26	0.625
Portal vein diameter (mm)		12.21 ± 1.99	11.27 ± 1.61	**0.068**
Portal vein flow rate (m/s)		0.18 ± .046	0.19 ± 0.08	0.580
Postoperative platelets (×10^9^/L)		316.37 ± 134.29	294.09 ± 119.54	0.536
Platelet differences between preoperative and postoperative (×10^9^/L)		251.16 ± 132.15	227.71 ± 107.07	0.486
Postoperative portal vein trunk diameter (mm)∗		11 (10~12) 11.44 ± 1.50	11 (10~12) 11.14 ± 1.82	0.367 (0.548)
Postoperative portal vein trunk flow rate (m/s)		0.19 ± 0.06	0.18 ± 0.06	0.545
Spleen volume (cm^3^)		711.44 ± 525.15	567.74 ± 246.92	0.272
Previous history of endoscopic treatment	No	**12 (63.2%)**	**28 (77.8%)**	**0.177**
	Yes	**7 (36.8%)**	**8 (22.2%)**	
Child-Pugh grade	A	15 (78.9%)	24 (68.6%)	0.416
	B	4 (21.1%)	11 (31.4%)	
Gender	Female	8 (42.1%)	12 (34.3%)	0.520
	Male	11 (57.9%)	23 (65.7%)	
Abdominal surgery history	No	17 (89.5%)	31 (88.6%)	1.0
	Yes	2 (10.5%)	4 (11.4%)	
Combined devascularization	No	7 (36.8%)	10 (25.7%)	0.392
	Yes	12 (63.2%)	26 (74.3%)	
Peripheral blood vessel dissection	No	12 (70.9%)	26 (74.3%)	0.392
	Yes	7 (36.8%)	10 (25.7%)	
Hypertension	No	**15 (78.9%)**	**33 (94.3%)**	**0.169**
	Yes	**4 (21.1%)**	**2 (5.7%)**	
Diabetes	No	14 (73.7%)	31 (88.6%)	0.251
	Yes	5 (26.3%)	4 (11.4%)	
Smoking	No	17 (89.5%)	31 (88.6%)	1.0
	Yes	2 (10.5%)	4 (11.4%)	
History of blood transfusion	No	9 (47.4%)	20 (57.1%)	0.492
	Yes	10 (52.6%)	15 (42.9%)	
Grade of splenomegaly ∗∗	0	**4 (21.1%)**	**16 (45.7%)**	**0.046** **(0.160)**
	I	**4 (21.1%)**	**6 (17.1%)**	
	II	**1 (5.3%)**	**4 (11.4%)**	
	III	**10 (52.6%)**	**9 (25.7%)**	
Grade of esophageal varices∗∗	No	1 (5.3%)	0 (0%)	0.229 (0.170)
	Mild	0 (0%)	1 (2.9%)	
	Moderate	1 (5.3%)	8 (22.9%)	
	Severe	17 (89.5%)	26 (74.3%)	
With gastric varices	No	7 (36.8%)	13 (37.1%)	0.983
	Yes	12 (63.2%)	22 (62.9%)	
Ascites	No	14 (73.7%)	26 (74.3%)	1
	Yes	5 (26.3%)	9 (25.7%)	
Hepatic encephalopathy	No	18 (94.7%)	35 (100%)	0.352
	Yes	1 (5.3%)	0	
Posthepatitic cirrhosis	No	8 (42.1%)	15 (42.9%)	0.957
	Yes	11 (57.9%)	20 (57.1%)	

∗*p* < 0.05 using the K–S test for data that were not normally distributed. Nonparametric tests were used to compare the groups. The results of Student's *t*-test are also included. ∗∗Graded variables. The rank-sum test was used to compare the groups, and the chi-square test results are also included.

**Table 4:** Multivariate analysis of thrombosis-related risk factors during follow-up (logistic regression: forward-LR method).

Risk factors	Regression coefficients	*p*	OR	95% confidence interval for OR
With hypertension	2.220	0.039	9.208	1.116~75.958
Grade of spleen enlargement	0.665	0.019	1.945	1.115~3.394
Creatinine	0.049	0.028	1.050	1.005~1.098
Constant	-5.483	0.004	0.004	

## Data Availability

The raw data used to support the findings of this study are restricted by the institution in order to protect patient privacy. If necessary, contact the corresponding author.
